# Effective Management of Refractory Paraneoplastic Vasculitis in Myelodysplastic Syndromes With Azacitidine, Prednisolone, and Azathioprine

**DOI:** 10.1155/crh/5914934

**Published:** 2025-11-06

**Authors:** Masamitsu Takaba, Kotaro Nakano, Akari Yoda, Yoshiro Otsuki, Shinya Fujisawa

**Affiliations:** ^1^Department of Hematology, Seirei Hamamatsu General Hospital, Hamamatsu, Japan; ^2^Department of Hematology, Iwata City Hospital, Iwata, Japan; ^3^Department of Pathology, Seirei Hamamatsu General Hospital, Hamamatsu, Japan

## Abstract

A 62-year-old man presented with left ear pain, sensorineural hearing loss, and high-grade fever. Peripheral blood tests revealed abnormal blood cells, prompting further investigation that led to a diagnosis of myelodysplastic syndromes (MDS) with paraneoplastic vasculitis. Since initial treatment with azacitidine (Aza) was insufficient, additional immunosuppressive therapy was required. The disease was effectively controlled with a combination of Aza, prednisolone, and azathioprine, with no relapse for 10 treatment courses. However, the disease later transformed into acute myeloid leukemia. This case highlights the efficacy and feasibility of additional treatment with steroids and azathioprine for refractory paraneoplastic vasculitis associated with MDS, while emphasizing the need for careful monitoring of leukemic progression under prolonged immunosuppression.

## 1. Introduction

Myelodysplastic syndromes (MDS) encompass a spectrum of clonal hematopoietic disorders characterized by the abnormal proliferation and differentiation of bone marrow cells into dysplastic blood cells. These dysplastic cells replace normal marrow, leading to ineffective hematopoiesis, cytopenia, and an increased risk of progression to acute myeloid leukemia (AML). In addition to hematopoietic dysfunction, MDS has been associated with various immune disorders, including vasculitis [1].

Paraneoplastic vasculitis is difficult to manage due to steroid dependence, with a high risk of relapse during tapering [2, 3]. Azacitidine (Aza), a demethylating agent commonly used for the treatment of MDS, has also been reported to be effective in certain autoimmune diseases associated with MDS [4, 5]. Aza monotherapy has been reported to be effective in some cases of MDS-associated paraneoplastic vasculitis [4, 6, 7].

Here, we describe a case of refractory MDS-associated paraneoplastic vasculitis that responded poorly to Aza monotherapy but achieved durable disease control with the addition of prednisolone and azathioprine.

## 2. Case Presentation

A 62-year-old man first developed left ear pain and hearing loss in October 2023. Despite receiving outpatient treatment at local clinics, his symptoms gradually worsened, and he subsequently developed right scleritis and headaches. As these symptoms persisted and intensified, he visited our hospital in November with marked erythema of the left external auditory canal and persistent hearing loss. Peripheral blood tests revealed abnormal blood cells. Although bone marrow smear showed no noticeable increase in dysplasia, G-banding and fluorescence in situ hybridization detected chromosomal abnormalities consistent with MDS, including Monosomy 7. Peripheral blood counts and bone marrow findings are summarized in Table 1. According to the WHO 2016 classification, the findings were consistent with MDS, unclassifiable, whereas under the WHO 2022 classification, they met the criteria for MDS with defining genetic abnormality. The patient's International Prognostic Scoring System (IPSS) and International Prognostic Scoring System-Revised (IPSS-R) indicated Intermediate-1 and Intermediate risk, respectively. The International Prognostic Scoring System-Molecular (IPSS-M) could not be evaluated because next-generation sequencing of the myeloid panel was not performed. Contrast-enhanced computed tomography revealed wall thickening in the right common, external, and internal carotid arteries, along with increased density in the surrounding adipose tissue. Antinuclear antibodies, MPO-ANCA, and PR3-ANCA were all negative. Genetic testing for *UBA1* mutations was performed to assess the possibility of VEXAS syndrome and detected no mutations. Based on these findings, the patient was diagnosed with MDS associated with paraneoplastic vasculitis. Although the patient's IPSS and IPSS-R scores suggested that immediate treatment for MDS was not necessary, severe symptoms of paraneoplastic vasculitis—including persistent headaches, fever, and hearing loss—necessitated intervention. Considering Aza's dual benefit for both MDS and its associated immune dysregulation, it was initiated instead of conventional immunosuppressive therapy, based on a previously published Japanese case report demonstrating its efficacy in a similar case [6].

**Figure 1:**
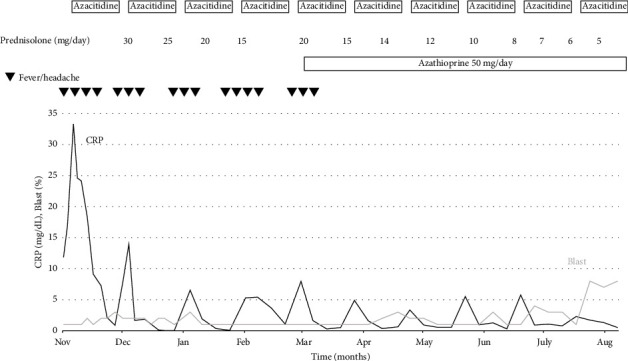
Clinical course showing symptoms, treatment, and trends in C-reactive protein (CRP) levels. The *x* axis represents time (months), and the *y* axis represents C-reactive protein (CRP) levels (mg/dL) and blast percentage (%) in peripheral blood. Episodes of fever and headache associated with vasculitis are indicated by (▼). Following the initiation of triple therapy with azacitidine, prednisolone, and azathioprine, both CRP levels and symptoms improved, and no further relapse was observed.

Figure 1 presents an overview of the patient's treatment course. After starting Aza, the patient's fever, headache, and C-reactive protein (CRP) levels temporarily improved. However, 2 weeks after initiating Aza, his symptoms and CRP levels worsened, indicating vasculitis relapse. As Aza monotherapy was insufficient, prednisolone (30 mg/day) was added, leading to improvements in symptoms and CRP levels. A second course of Aza was administered with gradual tapering of prednisolone. As with the first course, fever and CRP levels increased 2 weeks after starting Aza. The third and fourth courses showed patterns similar to those of the first two courses, with transient improvement followed by relapse. Azathioprine (50 mg/day) was added from the fifth course to facilitate steroid tapering as illustrated in Figure 1. Since then, no clinical signs of vasculitis relapse, including headache and fever, have been observed under this triple therapy regimen. The tapering of the prednisolone was continued gradually. Finally, vasculitis was effectively controlled with triple therapy over 10 courses of Aza. No adverse events, including infections, were observed during the treatment period. After 10 courses of treatment, however, MDS progressed to AML, necessitating the initiation of AML-targeted chemotherapy.

## 3. Discussion

In this case, while Aza monotherapy was ineffective in controlling the patient's paraneoplastic vasculitis, the combination of Aza, prednisolone, and azathioprine led to a favorable therapeutic response. Unlike previous reports in which Aza monotherapy successfully controlled the disease, our case underscores the pivotal role of combination therapy with Aza, prednisolone, and azathioprine in managing refractory paraneoplastic vasculitis associated with MDS.

The relationship between MDS and autoimmune diseases is complex, suggesting that each may influence the development and progression of the other. Studies have shown that 10%–30% of patients with MDS have systemic inflammatory or autoimmune diseases [8, 9]. Many of these autoimmune diseases are diagnosed within 1 year of MDS onset [10]. When MDS develops, inflammatory activity in the bone marrow microenvironment is induced, which may directly or indirectly trigger systemic inflammation and autoimmune diseases [1]. Therefore, treatments for MDS may also be effective against its associated autoimmune diseases [1].

Demethylating agents such as Aza have demonstrated efficacy in the treatment of MDS-associated autoimmune disorders, including autoimmune hemolytic anemia, Behçet's disease, and other paraneoplastic inflammatory conditions [8, 11, 12]. However, reports on the use of Aza for MDS-associated paraneoplastic vasculitis remain limited [4, 6, 7]. Previous reports suggest that Aza monotherapy can achieve favorable disease control in some cases. The response to Aza may depend on the underlying pathophysiology of MDS-associated vasculitis. In refractory cases, the addition of steroids and azathioprine to Aza has been suggested as an effective therapeutic strategy. No adverse events, including infections, were reported during treatment, demonstrating the safety and feasibility of this regimen in treating refractory vasculitis. However, since this was a single case report, it is difficult to determine to what extent Aza itself contributed to the improvement of vasculitis apart from the effects of concomitant immunosuppressive therapy. In cases with a strong autoimmune component, Aza alone may not provide sufficient immunosuppression, necessitating additional therapy with steroids and azathioprine. At the same time, leukemic transformation may occur earlier than expected for the IPSS or IPSS-R risk categories, possibly due to prolonged immunosuppression that weakens antitumor immunity. Thus, careful monitoring and the use of molecular-based risk assessment, such as IPSS-M, are warranted to better guide prognosis and treatment decisions.

In conclusion, although Aza monotherapy has been effective in some cases of MDS-associated paraneoplastic vasculitis, its efficacy may be limited in patients with severe or multiorgan vasculitic involvement. In such cases, the addition of steroids and immunosuppressants may be beneficial, but the treatment strategy should be determined carefully, taking into account the severity of vasculitis and the potential risk of leukemic transformation under prolonged immunosuppression. More case reports are needed to optimize the management strategies for MDS-associated paraneoplastic vasculitis.

## Figures and Tables

**Table 1:** Peripheral blood counts and bone marrow findings at diagnosis.

*Blood cell count*	
WBC	12,680 *μ*L
Seg	37.0%
Mono	39.0%
Lympho	18.0%
Eosino	0.0%
Baso	0.0%
Myelo	1.0%
Meta	3.0%
Blast	1.0%
RBC	491 × 104 *μ*L
Hb	12.7 g/dL
Hct	39.8%
Plt	29.9 × 104 *μ*L
MCV	81.1 fL

*Coagulation*	
PT	11.6 s
PT-INR	1.07
APTT	27.9 s
Fib	372 mg/dL

*Biochemistry*	
TP	6.9 g/dL
Alb	3.8 g/dL
T-Bil	1.2 mg/dL
AST	14 U/L
ALT	23 U/L
LDH	143 U/L
BUN	9 mg/dL
Cre	0.68 mg/dL
UA	5.7 mg/dL
Na	138 mEq/L
K	4.2 mEq/L
Cl	99 mEq/L
IgG	1138 mg/dL
IgA	204 mg/dL
IgM	79 mg/dL
CRP	11.8 mg/dL
ANA	< 40 index
MPO-ANCA	< 1.0 U/mL
PR3-ANCA	< 1.0 U/mL

*Bone marrow smear*	
NCC	20.2 × 104 *μ*L
Megk	30.0 *μ*L
Myeloid series	
Myeloblast	4.0%
Promyelo	6.0%
Myelo	19.4%
Meta	5.4%
Stab	8.0%
Seg	12.2%
Eosino	0.0%
Baso	0.6%
Erythroid series	
ProE	1.0%
Baso	3.0%
Poly	19.6%
Ortho	0.4%
Lymphocyte	6.2%
Plasma cell	0.0%
Monocyte	4.4%

Abbreviations: ANA, antinuclear antibody; Megk, megakaryocyte; MPO-ANCA, myeloperoxidase antineutrophil cytoplasmic antibody; NCC, nucleated cell count; PR3-ANCA, proteinasae-3-antineutrophil cytoplasmic antibody.

## Data Availability

Data sharing is not applicable to this article as no new data were created or analyzed in this study.
